# The relationship between blood glucose and clinical outcomes after extracorporeal circulation: a retrospective cohort study

**DOI:** 10.3389/fcvm.2025.1480163

**Published:** 2025-03-31

**Authors:** Li Li, Rui Li, Yi-Jiang Liu, Zhan Wang, Xin Chen, Lin-Xi Xu, Zhi-Huang Chen, Jia-Cheng Xu, Zhong-Gui Shan

**Affiliations:** ^1^ Department of Cardiovascular Surgery, The First Affiliated Hospital of Xiamen University, School of Medicine, Xiamen University, Xiamen, China; ^2^ The School of Clinical Medicine, Fujian Medical University, Fuzhou, China

**Keywords:** extracorporeal circulation, cardiac surgery, blood glucose, MIMIC-IV, ICU

## Abstract

**Background:**

Postoperative blood glucose levels significantly impact outcomes in cardiac surgery patients undergoing extracorporeal circulation (ECC) auxiliary to open heart surgery. While hypoglycemia and hyperglycemia are known risk factors for adverse outcomes, the optimal glycemic range for patients undergoing ECC remains unclear. This research examined the relationship between blood glucose levels and 90-day mortality in this high-risk group.

**Methods:**

The data for this research were obtained from the Medical Information Mart for Intensive Care-IV database version 2.2(MIMIC-IV 2.2), including 4,033 patients who underwent ECC-assisted open-heart surgery. Patients were classified into quartiles based on blood glucose values measured within a 24 h period following admission to the ICU. The study's primary outcome was the 90-day mortality, and the duration of hospital and ICU stays were considered secondary outcomes. Kaplan–Meier survival analysis, Multivariate Cox regression models, smooth curve fitting, Restricted Cubic Spline (RCS) curve, and subgroup analyses were conducted to evaluate the relationship between blood glucose levels and patient outcomes.

**Results:**

Higher blood glucose levels were significantly related to increased 90-day mortality. The analysis revealed the nonlinear relation between blood glucose and 90 days mortality, with an inflection point at 119 mg/dl (*P* < 0.05). Patients with blood glucose levels above this threshold had a markedly higher risk of mortality. Additionally, elevated blood glucose was associated with prolonged hospitalization and ICU stays.

**Conclusion:**

Elevated postoperative blood glucose values were related to an increased 90-day mortality in patients who underwent ECC. When blood glucose levels exceeded 119 mg/dl, blood glucose levels were positively associated with 90-day postoperative mortality.

## Introduction

The assistance of extracorporeal circulation (ECC) auxiliary to open heart surgery is often required to perform most cardiac surgical procedures, with millions of patients worldwide undergoing this operation annually (1). The relationship between blood glucose values and clinical outcomes in patients undergoing ECC has garnered increasing attention recently. Hyperglycemia, a common occurrence in ICU patients (2), is recognized to be linked to unfavorable consequences across various medical conditions, including cardiac surgery. Studies analyzing data from over eight million patients have found that approximately 8% of these patients have diabetes, making it the second most prevalent comorbidity in the perioperative period (3). The physiological stress of surgery, combined with the use of ECC, can exacerbate hyperglycemia, which in turn may lead to a cascade of complications such as infection, organ dysfunction, and increased mortality. Suboptimal postoperative blood glucose control can exacerbate the patient's condition, increase the economic burden on the patient, and place additional financial strain on health insurance systems (4).

In recent years, the mortality rate of cardiac surgery has decreased due to advancements in surgical techniques and perioperative management (5). However, cardiac surgery remains a highly risky procedure. The relatively high mortality rate associated with cardiac surgery is largely attributable to its complex nature and the critical condition of the patients (6). Consequently, clinicians are increasingly emphasizing the assessment of mortality risk in cardiac surgery. At the same time, blood glucose control in patients with ECC is also important, especially in elder patients (7), as the majority of patients undergoing cardiac surgery are elderly. The unique pathophysiological changes induced by ECC, including altered blood flow, inflammatory responses, and coagulation disturbances, may modify how blood glucose levels and patient outcomes are related. Recently, hemolysis has been associated with insulin degradation in a simulated ECC model, which may be a potential cause of intraoperative hyperglycemia (8). Additionally, surgical trauma can induce insulin resistance, further contributing to postoperative hyperglycemia (9). Postoperative hyperglycemia may increase the probability of infection in the patient (10). Conversely, patients with low blood glucose levels post-surgery, especially those who are fasting following ECC in the ICU, are at higher risk of hypoglycemia and adverse events (11, 12). Therefore, understanding the nuances of glycemic control in this setting is crucial for optimizing patient management and improving survival rates.

Previous studies have reported mixed findings, with some suggesting that tight glycemic control may reduce complications (13), while others caution against the risks of hypoglycemia (14). These inconsistencies highlight the need for a nuanced approach that considers the risks and benefits of different glycemic targets. Given the complex interplay between hyperglycemia and ECC, there is a pressing need to elucidate the optimal range of blood glucose levels that can minimize postoperative complications and enhance recovery.

This research investigates the connection between glucose levels and the 90-day mortality in patients undergoing ECC. By analyzing data from a comprehensive cohort, we seek to provide evidence-based recommendations for blood glucose management in this high-risk population, thereby contributing to the ongoing efforts to refine perioperative care in cardiac surgery.

## Methods

### Data source

The data for this research were obtained from the Medical Information Mart for Intensive Care-IV database version 2.2 (MIMIC-IV) (15). The MIMIC-IV is a publicly available critical care database developed by the Massachusetts Institute of Technology (MIT), Cambridge, MA, USA. It includes de-identified health information on 73,181 patients. Admitted to the ICU at Beth Israel Deaconess Medical Center (BIDMC) between 2008 and 2019. The data includes demographic details, vital signs, laboratory test results, and prescribed medications.

This study was conducted in compliance with ethical guidelines, and the demand for informative consent was waived due to the utilization of de-identified data. One author, Li Li, accessed the MIMIC-IV and performed data extraction after completing the necessary data use and protection training (certification number 13369960). The data extraction complied with the Health Insurance Portability and Accountability Act (HIPAA) Safe Harbor rules, ensuring that all patient-identifying information was removed to maintain privacy and confidentiality (16).

### Study populations

This research included 4,033 patients who underwent ECC and were subsequently admitted to the ICU. Patients' diagnoses were based on the International Classification of Diseases, ninth revision (ICD-9), and tenth revision (ICD-10). Study populations were classified into four groups in quartiles based on their blood glucose values during the first ICU admission after ECC. Exclusion criteria were age under 18 years, staying in the ICU for less than 3 h, and not having an initial admission to the ICU. Patients with missing key data, such as blood glucose values or 90-day follow-up visit records, were excluded.

### Variables

The primary outcome of this research was 90-day mortality, which was interpreted as any death that happened within 90 days following being admitted to the ICU. This covered both in- and out-of-hospital deaths. The primary risk exposure was blood glucose level, which was obtained from the first arterial blood gas measurement during the initial 24 h of ICU stay after cardiac surgery with ECC. Covariates included age, sex, body mass index (BMI); vital signs at the moment of admission to the ICU (such as heart rate, MAP, SBP, DBP, and peripheral oxygen saturation (SpO_2_); laboratory indicators [potassium, creatinine, hemoglobin, neutrophils, monocytes, lymphocytes, white blood cell (WBC), and glucose]; comorbidities (myocardial infarction(MI), congestive heart failure(CHF), peripheral vascular disease(PVD), cerebrovascular disease(CVD), chronic pulmonary disease(CPD), diabetes(DM), renal disease(RD), cancer); and prognostic information, mechanical ventilation hours, the lengths of stay in a hospital (LOS hospital) and ICU (LOS ICU).

### Outcomes

The primary outcome was the 90-day mortality, and the duration of hospital and ICU stays were considered secondary outcomes.

### Statistical analysis

In the statistical process, we assumed that missing covariate data occurred randomly, and therefore, multiple interpolation was used to compensate for missing covariate data. The study population's baseline characteristics were compiled using descriptive statistics. The continuous variable is displayed as mean ± standard deviation (SD) if it has a normal distribution and as median (interquartile range, or IQR) if it does not. Categorical variables can be expressed by percentages and frequencies. Based on blood glucose levels, the study population was split into quartiles, and variations in baseline characteristics across quartiles were evaluated, when applicable, using ANOVA or chi-square testing (17, 18).

To compare 90-day mortality among blood glucose levels quartiles, Kaplan–Meier survival curves were created, and the log-rank test was used to establish their statistical significance. To determine the relationship between blood glucose levels and the patients' 90-day postoperative prognosis, we performed smoothed curve fitting, and Restricted Cubic Spline (RCS) curve analyses. Multivariate Cox regression models were built to assess the association between blood glucose values in the quartiles and the 90-day mortality. The multivariate model adjusted for potential confounders identified *a priori* based on clinical relevance and previous literature, including age, sex, race, BMI, heart rate, MAP, SpO2, and comorbidities (such as MI, CHF, PVD, CPD, DM, RD, cancer). We used IBM SPSS Statistics Version 27.0 (IBM Corp, Armonk, NY), EmpowerStats (Boston, MA) and R software version 4.3.3 (Vienna, Austria) in the RStudio setting to conduct statistical analysis. When the *P* value was less than 0.05, it was considered statistically significant.

## Results

### Patient population

**Figure 1:**
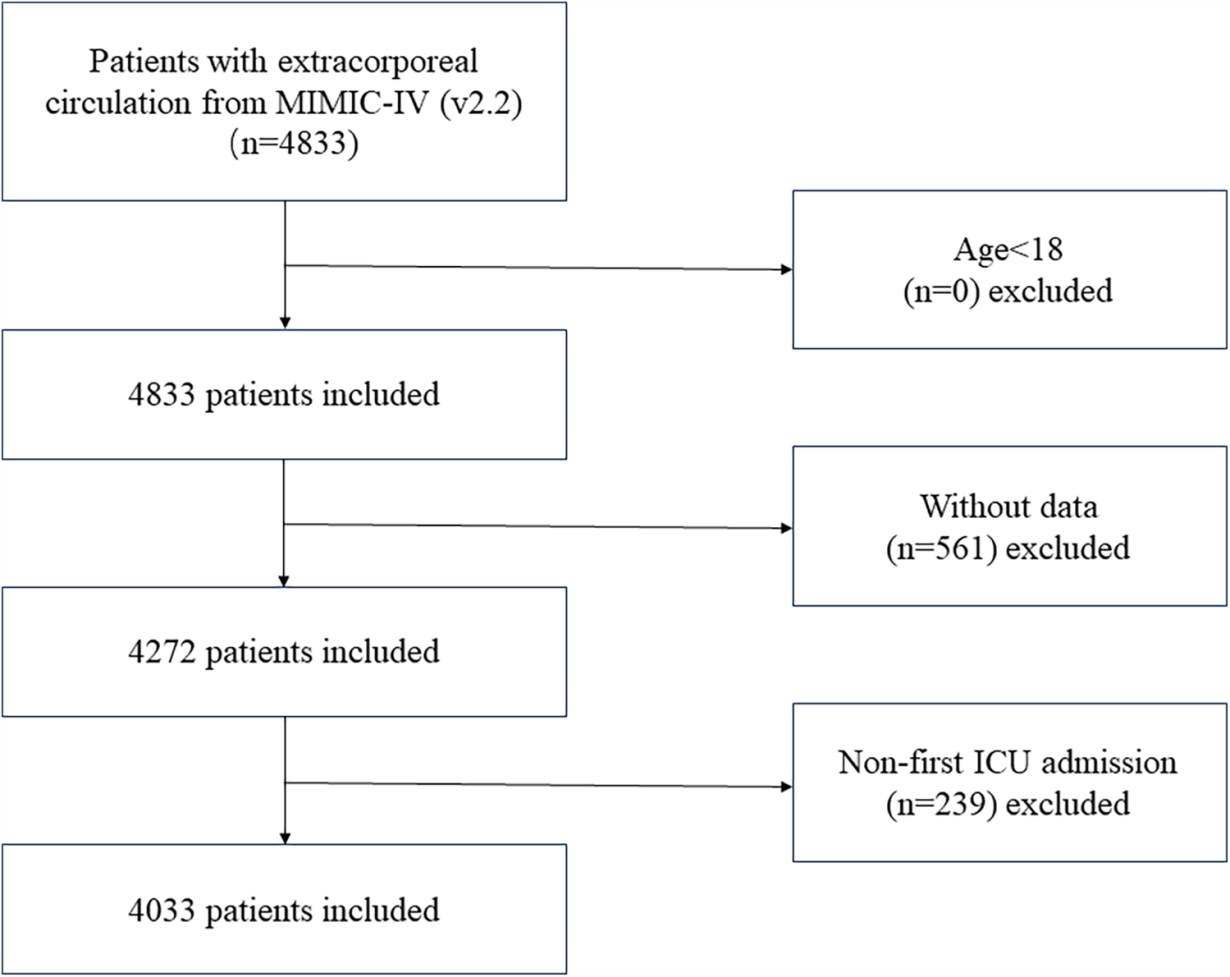
Flowchart showing the criteria for inclusion and exclusion.

Based on the 9th edition of the International Classification of Diseases (ICD-9), we extracted data related to ECC procedures from the MIMIC-IV database, with the corresponding ICD code “3961”. The study's initial sample consisted of 4,833 individuals who had cardiopulmonary bypass surgery after cardiac surgery. Due to missing baseline data or lack of follow-up records, 452 patients were excluded. Additionally, because glucose values were lacking, 109 participants were removed, and 239 patients were excluded as they were not first-time ICU admissions (Including patients requiring secondary surgery and patients with severe trauma who have failed resuscitation with ECC). Consequently, the final cohort comprised 4,033 patients Figure 1.

### Baseline characteristics of participants

The quartiles were used to split the study population into four groups according to the blood glucose values recorded on the first day of the ICU stay. The mean age of the population included in the study was 67.8 ± 11.9 years, and 69.5% were male. The distribution of baseline characteristics for each group of patients was as follows Table 1: (Q1) 996 patients in the group with glucose ≤102 mg/dl, (Q2) 986 patients in the group with 102–117 mg/dl, (Q3) 1,002 patients in the group with 117–134 mg/dl, and (Q4)1,049 patients in the group with glucose >134 mg/dl. Q4 (highest glucose quartile: 166.6 ± 49.2 mg/dl) demonstrated markedly elevated 90-day mortality (4.8% vs. 1.9%–2.3% in Q1-Q3, *P* < 0.001), accompanied by progressive increases in BMI (*P* < 0.001), SBP (*P* = 0.003), creatinine (*P* < 0.001), and WBC count (*P* < 0.001), suggesting a gradient of metabolic dysregulation and systemic inflammation proportional to glucose levels. Clinically, Q4 exhibited higher prevalence of DM (43.09% vs. 25.76%–30.92%, *P* < 0.001), RD (17.83% vs. 11.16%–13.15%, *P* < 0.001), and cardiometabolic comorbidities (MI: 32.51% vs. 22.95%–24.65%; CHF: 32.41% vs. 23.55%–24.75%, *P* < 0.001), which collectively may drive adverse outcomes. Prolonged mechanical ventilation (*P* < 0.001) and ICU or hospital stays (*P* < 0.001) in Q4 further indicate greater postoperative complexity.

**Table 1:** Baseline characteristics of the study participants by quartiles of blood glucose.

Blood Glucose Quartile	Q1 (*n* = 996)	Q2 (*n* = 986)	Q3 (*n* = 1,002)	Q4 (*n* = 1,049)	*P*-value
Age, years	68.35 ± 12.46	67.64 ± 12.07	67.40 ± 12.16	67.84 ± 11.33	0.156
Gender, sex					0.071
Male, *n* (%)	670 (67.27%)	711 (72.11%)	709 (70.76%)	716 (68.26%)	
Female, *n* (%)	326 (32.73%)	275 (27.89%)	293 (29.24%)	333 (31.74%)	
Race					0.719
Asian, *n* (%)	23 (2.3%)	23 (2.3%)	21 (2.1%)	19 (1.8%)	
Black, *n* (%)	31 (3.1%)	44 (4.5%)	35 (3.5%)	40 (3.8%)	
White, *n* (%)	761 (76.4%)	766 (77.7%)	787 (78.5%)	820 (78.2%)	
Unknown, *n* (%)	144 (14.5%)	119 (12.1%)	119 (11.9%)	139 (13.3%)	
Other, *n* (%)	37 (3.7%)	34 (3.4%)	40 (4.0%)	31 (3.0%)	
BMI, kg/m^2^	30.16 ± 5.73	30.88 ± 6.07	31.44 ± 6.40	31.84 ± 6.49	<0.001
Heart Rate, bpm	80.76 ± 10.40	80.75 ± 11.23	80.97 ± 10.91	81.81 ± 12.41	0.294
MAP (mmHg)	70.56 ± 12.06	71.04 ± 11.31	71.45 ± 11.99	71.89 ± 13.56	0.083
SBP (mmHg)	110.65 ± 17.90	111.06 ± 17.25	112.39 ± 18.27	113.40 ± 20.38	0.003
DBP (mmHg)	58.56 ± 12.55	59.02 ± 11.42	59.36 ± 12.24	59.18 ± 13.20	0.241
SpO_2_ (%)	96.57 ± 4.82	96.50 ± 5.12	96.36 ± 5.30	95.62 ± 7.43	0.004
Blood Glucose (mg/dl)	89.8 ± 10.0	109.1 ± 4.3	124.6 ± 4.9	166.6 ± 49.2	<0.001
Potassium (mmol/L)	4.21 ± 0.50	4.19 ± 0.51	4.21 ± 0.50	4.25 ± 0.55	0.048
Creatinine (mg/dl)	0.99 ± 0.77	0.94 ± 0.53	0.97 ± 0.82	1.10 ± 0.88	<0.001
Hemoglobin (g/dl)	9.88 ± 2.09	10.21 ± 2.06	10.01 ± 1.97	10.25 ± 2.14	<0.001
Neutrophils (10^9^/L)	9.30 ± 4.34	9.52 ± 5.36	9.82 ± 4.26	9.77 ± 4.90	0.078
Monocytes (10^9^/L)	0.39 ± 0.31	0.36 ± 0.26	0.38 ± 0.28	0.39 ± 0.24	0.018
Lymphocytes (10^9^/L)	2.36 ± 6.46	1.97 ± 1.02	2.01 ± 1.00	1.95 ± 1.75	0.130
WBCs (10^9^/L)	11.69 ± 5.30	11.88 ± 5.16	12.44 ± 5.67	12.49 ± 6.17	<0.001
Ventilation Hours (hours)	56.29 ± 97.69	51.19 ± 91.87	58.34 ± 96.98	76.74 ± 118.83	<0.001
LOS ICU (days)	2.90 ± 4.41	2.58 ± 3.27	2.83 ± 3.62	3.55 ± 4.56	<0.001
LOS hospital (days)	8.9 ± 7.3	8.4 ± 5.9	8.3 ± 5.6	9.8 ± 7.0	<0.001
Myocardial Infarct					<0.001
No, *n* (%)	767 (77.01%)	743 (75.35%)	772 (77.05%)	708 (67.49%)	
Yes, *n* (%)	229 (22.99%)	243 (24.65%)	230 (22.95%)	341 (32.51%)	
Congestive Heart Failure					<0.001
No, *n* (%)	751 (75.40%)	742 (75.25%)	766 (76.45%)	709 (67.59%)	
Yes, *n* (%)	245 (24.60%)	244 (24.75%)	236 (23.55%)	340 (32.41%)	
Peripheral Vascular Disease					0.012
No, *n* (%)	840 (84.34%)	846 (85.80%)	837 (83.53%)	845 (80.55%)	
Yes, *n* (%)	156 (15.66%)	140 (14.20%)	165 (16.47%)	204 (19.45%)	
Cerebrovascular Disease					0.384
No, *n* (%)	882 (88.55%)	872 (88.44%)	906 (90.42%)	943 (89.90%)	
Yes, *n* (%)	114 (11.45%)	114 (11.56%)	96 (9.58%)	106 (10.10%)	
Chronic Pulmonary Disease					0.688
No, *n* (%)	765 (76.81%)	737 (74.75%)	757 (75.55%)	803 (76.55%)	
Yes, *n* (%)	231 (23.19%)	249 (25.25%)	245 (24.45%)	246 (23.45%)	
Diabetes					<0.001
No, *n* (%)	688 (69.08%)	732 (74.24%)	700 (69.86%)	597 (56.91%)	
Yes, *n* (%)	308 (30.92%)	254 (25.76%)	302 (30.14%)	452 (43.09%)	
Renal Disease					<0.001
No, *n* (%)	865 (86.85%)	876 (88.84%)	872 (87.03%)	862 (82.17%)	
Yes, *n* (%)	131 (13.15%)	110 (11.16%)	130 (12.97%)	187 (17.83%)	
Cancer					0.678
No, *n* (%)	963 (96.69%)	960 (97.36%)	977 (97.50%)	1,017 (96.95%)	
Yes, *n* (%)	33 (3.31%)	26 (2.64%)	25 (2.50%)	32 (3.05%)	
90-Day Mortality					<0.001
No, *n* (%)	976 (98.0%)	963 (97.7%)	983 (98.1%)	999 (95.2%)	
Yes, *n* (%)	20 (2.0%)	23 (2.3%)	19 (1.9%)	50 (4.8%)	

If quantitative variables are normally distributed, they are presented as mean ± standard deviations; if they are abnormally distributed, they are presented as median (25th percentile, 75th percentile).

BMI, body mass index; MAP, mean artery pressure; SBP, systolic blood pressure; DBP, diastolic blood pressure; WBC, white blood cell; LOS ICU, length of stay in ICU; LOS hospital, length of stay at the hospital.

### The results of univariate analysis

The univariate analysis evaluated associations between various clinical parameters and 90-day mortality in patients undergoing ECC (Table 2). Age demonstrated a significant increase in mortality risk (HR 1.02, 95% CI 1.01–1.04, *P* = 0.006), as did sex (HR: 1.49, 95% CI: 1.02–2.16, *P* = 0.038). Key physiological markers linked to higher mortality included elevated heart rate (HR: 1.03, 95% CI: 1.01–1.04, *P* < 0.001), lower DBP (HR: 0.98, 95% CI: 0.96–0.99, *P* = 0.006), reduced SpO₂ (HR: 0.93, 95% CI: 0.90–0.96, *P* < 0.001), hyperkalemia (HR: 1.47,95% CI: 1.07–2.02, *P* = 0.019), and elevated creatinine (HR: 1.25, 95% CI: 1.18–1.34, *P* < 0.001). The HR for prolonged ventilation was 1, indicating no difference in risk between groups (HR: 1.00, 95% CI: 1.00–1.00, *P* < 0.001). Longer ICU stays and hospital stays (HR: 1.08 and 1.10, 95% CI: 1.07–1.10 and 1.10–1.10, respectively, *P* < 0.001) were also significant influencing factors.

**Table 2:** The results of univariate analysis.

Exposure	Overall	90-Day Mortality HR (95% CI)	*P*-value
Age	67.83 ± 11.99	1.02 (1.01, 1.04)	0.006
Sex			
Male	2,806 (69.58%)	Reference	
Female	1,227 (30.42%)	1.49 (1.02, 2.16)	0.038
BMI	31.09 ± 6.26	1.00 (0.97, 1.03)	0.970
Heart Rate	81.08 ± 11.28	1.03 (1.01, 1.04)	<0.001
SBP	111.78 ± 18.53	1.00 (0.99, 1.01)	0.503
DBP	59.01 ± 12.35	0.98 (0.96, 0.99)	0.006
MAP	71.20 ± 12.26	0.98 (0.96, 1.00)	0.031
SpO_2_	99.05 ± 2.40	0.93 (0.90, 0.96)	<0.001
Potassium	4.22 ± 0.52	1.47 (1.07, 2.02)	0.019
Creatinine	1.00 ± 0.76	1.25 (1.18, 1.34)	<0.001
Hemoglobin	10.07 ± 2.07	0.93 (0.85, 1.02)	0.137
Platelet	169.86 ± 68.01	1.00 (1.00, 1.00)	0.879
Neutrophils	9.63 ± 4.73	1.02 (0.97, 1.07)	0.388
Monocytes	0.38 ± 0.27	1.90 (0.92, 3.93)	0.084
Lymphocytes	2.06 ± 3.28	0.64 (0.46, 0.90)	0.009
WBC	12.15 ± 5.60	1.02 (0.99, 1.05)	0.135
Ventilation Hours	61.05 ± 103.42	1.00 (1.00, 1.00)	<0.001
LOS ICU	2.98 ± 4.04	1.08 (1.07, 1.10)	<0.001
LOS Hospital	8.9 ± 6.5	1.10 (1.10, 1.10)	<0.001
Blood Glucose Quartile			
Q1	996 (24.7%)	Reference	
Q2	986 (24.4%)	1.20 (0.6, 2.1)	0.625
Q3	1,002 (24.8%)	0.90 (0.5, 1.8)	0.851
Q4	1,049 (26.0%)	2.40 (1.4, 4.1)	<0.001
Blood Glucose	123.1 ± 38.5	1.0 (1.0, 1.0)	<0.001
Myocardial Infarct			
No	2,990 (74.14%)	Reference	
Yes	1,043 (25.86%)	1.88 (1.28, 2.74)	0.001
Congestive Heart Failure			
No	2,968 (73.59%)	Reference	
Yes	1,065 (26.41%)	3.16 (2.18, 4.57)	<0.001
Peripheral Vascular Disease			
No	2,968 (73.59%)	Reference	
Yes	1,065 (26.41%)	2.66 (1.80, 3.94)	<0.001
Cerebrovascular Disease			
No	3,603 (89.34%)	Reference	
Yes	430 (10.66%)	2.44 (1.57, 3.81)	0.001
Chronic Pulmonary Disease			
No	3,062 (75.92%)	Reference	
Yes	971 (24.08%)	1.70 (1.15, 2.50)	0.007
Diabetes			
No	2,717 (67.37%)	Reference	
Yes	1,316 (32.63%)	1.29 (0.88, 1.89)	0.1926
Renal Disease			
No	3,475 (86.16%)	Reference	
Yes	558 (13.84%)	3.27 (2.21, 4.83)	<0.001
Cancer			
No	3,917 (97.12%)	Reference	
Yes	116 (2.88%)	0.61 (0.15, 2.47)	0.473

BMI, body mass index; MAP, mean artery pressure; SBP, systolic blood pressure; DBP, diastolic blood pressure; WBC, white blood cell; LOS ICU, length of stay in ICU; LOS hospital, length of stay at the hospital; HR, hazard ratio; CI, confidence interval.

HR values less than 1 indicates a reduced risk of 90-day mortality.

Among comorbidities, MI (HR:1.88, 95% CI: 1.28–2.74, *P* = 0.001), CHF (HR: 3.16, 95% CI 2.18–4.57, *P* < 0.001), PVD (HR: 2.66, 95% CI: 1.80–3.94, *P* < 0.001), CVD (HR: 2.44,95% CI:1.57–3.81, *P* = 0.001), CPD (HR: 1.70, 95% CI: 1.15–2.50, *P* = 0.007), and RD (HR: 3.27, 95% CI: 2.21–4.83, *P* < 0.001) markedly increased mortality risk. Blood glucose levels in the highest quartile (Q4) showed a pronounced association with mortality (HR: 2.40, 95% CI: 1.4–4.1 vs. Q1, *P* < 0.001).

### The results of subgroup analyses

Subgroup comparisons were used to test whether the relationship between blood glucose values and 90-day mortality was stable across subgroups (Table 3). There were no obvious interactions found between the glucose and age, sex, BMI, LOS ICU, ventilation hours, CVD, CHF, PVD, CPD, RD, and cancer (*P* for interaction >0.05). We observed evidence of an interaction between glucose and LOS hospital, MI, and DM (*P* for interaction <0.05). The results indicate that the impact of blood glucose levels on 90-day postoperative mortality differs significantly between non-MI and MI patients and between non-DM and DM patients.

**Table 3:** The results of subgroup analysis.

Sub-Group	Total	Effect size (95% CI)	*P*-Value	*P* for Interaction*
Age Tertile				0.44
≤63	1,348	1.01 (1.01, 1.01)	<0.001	
63–73	1,339	1.01 (1.00, 1.01)	0.019	
≥73	1,346	1.01 (1.00, 1.02)	0.001	
Sex				0.26
Male	2,806	1.01 (1.01, 1.01)	<0.001	
Female	1,227	1.01 (1.00, 1.01)	0.003	
BMI Tertile				0.10
≤28	1,171	1.01 (1.00, 1.01)	0.023	
28–34	1,182	1.01 (1.01, 1.02)	<0.001	
>34	1,183	1.01 (1.00, 1.01)	0.043	
LOS ICU	4,033	1.01 (1.00, 1.01)	<0.001	0.57
LOS Hospital	4,033	1.01 (1.00, 1.01)	<0.001	<0.01
Ventilation hours Tertile				0.81
Low	1,324	1.01 (1.00,1.02)	0.028	
Middle	1,320	1.01 (1.00,1.01)	0.017	
High	1,326	1.01 (1.00,1.01)	0.001	
Myocardial infarct				0.01
No	2,990	1.01 (1.01,1.02)	<0.001	
Yes	1,043	1.00 (1.00,1.01)	0.038	
Cerebrovascular Disease				0.11
No	3,603	1.01 (1.01, 1.01)	<0.001	
Yes	430	1.00 (0.99, 1.01)	0.538	
Congestive Heart Failure				0.08
No	2,968	1.01 (1.01, 1.02)	<0.001	
Yes	1,065	1.01 (1.00, 1.01)	0.005	
Peripheral Vascular Disease				0.92
No	3,368	1.01 (1.00, 1.01)	<0.001	
Yes	665	1.01 (1.00, 1.01)	0.002	
Chronic Pulmonary Disease				0.52
No	3,062	1.01 (1.01, 1.01)	<0.001	
Yes	971	1.01 (1.00, 1.01)	0.046	
Diabetes				<0.01
No	2,717	1.02 (1.01, 1.02)	<0.001	
Yes	1,316	1.00 (1.00, 1.01)	0.629	
Renal Disease				0.13
No	3,475	1.01 (1.01, 1.01)	<0.001	
Yes	558	1.00 (1.00, 1.01)	0.054	
Cancer				0.77
No	3,917	1.01 (1.01, 1.01)	<0.001	
Yes	116	1.00 (0.97, 1.04)	0.829	

*P* for Interaction*: It is a statistical metric used to detect whether there are significant differences in effects across different subgroups. It indicates whether there is sufficient evidence in the statistical analysis to show that the impact of variables on the outcome varies between subgroups when assessing the interaction effects between two or more variables.

### Association between postoperative blood glucose and outcomes

**Figure 2:**
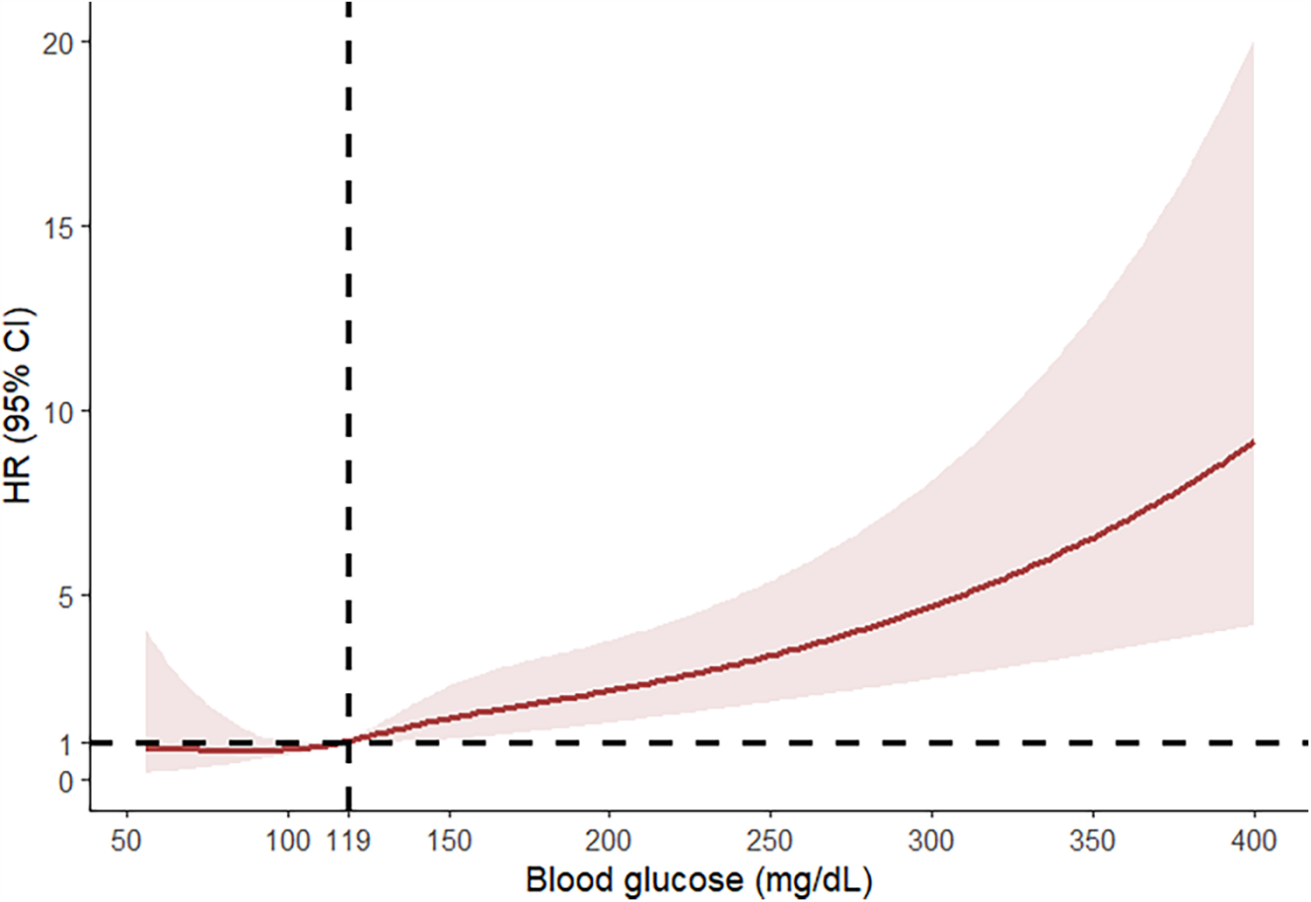
RCS curve analysis on the relationship between blood glucose levels and 90-day mortality.

Among 4,033 patients with high glucose levels (Q4) who underwent ECC, the mortality rates at postoperative 30 days to one year were significantly higher compared to the low blood glucose levels groups (Q1–Q3) (Supplementary Table S1) (*P* < 0.05). This study discovered a nonlinear connection between blood glucose levels and 180-day mortality (after adjusting age, sex, race, BMI, heart rate, MAP, SpO_2_, MI, CHF, CVD, CPD, DM, RD, and cancer).We analyzed the relationship between blood glucose levels and 90-day mortality using an RCS curve (Figure 2). The results indicated that when blood glucose levels were below 119 mg/dl, the HR was less than 1, whereas when blood glucose levels exceeded 119 mg/dl, the HR was greater than 1. This finding demonstrates that blood glucose levels above 119 mg/dl were significantly associated with an increased risk of 90-day mortality.

A multivariate Cox regression model assessed the associations between blood glucose levels and 90-day mortality (Table 4). In the non-adjusted model, elevated blood glucose levels were significantly associated with an increased risk of 90-day mortality (HR: 1.01, 95% CI: 1.01–1.01, *P* < 0.01). Increased blood glucose levels in the incompletely adjusted (age, sex, race, and BMI) and fully adjusted (sex, age, race, BMI, heart rate, MAP, SpO_2_, MI, CHF, PVD, CVD, CPD, DM, RD, and cancer) models were associated with an increased risk of 90-day mortality in the study population. For sensitivity analysis, this study used the continuous variable blood glucose as a quartile and showed that in the high glucose group (Q4), the 90-day mortality was considerably greater compared to the low glucose group (Q1). This finding was consistent across the non-adjusted model in the high blood glucose level group (Q4) (HR: 2.42, 95% CI: 1.44–4.06), minimally adjusted model (HR: 2.51, 95% CI: 1.40–4.48), and fully adjusted model (HR: 2.24, 95% CI: 1.24–4.03) (*P* < 0.05).

**Table 4:** Multivariate Cox regression to assess the association between glucose and 90-day mortality.

Variables	Non-adjusted	Model I	Model II
	HR (95% CI) *P* value	HR (95% CI) *P* value	HR (95% CI) *P* value
Blood Glucose	1.01 (1.01, 1.01) < 0.001	1.01 (1.01, 1.01) < 0.001	1.01 (1.00, 1.01) < 0.001
Blood Glucose Quartile			
Q1	Reference	Reference	Reference
Q2	1.16 (0.64, 2.11) 0.625	1.28 (0.66, 2.49) 0.470	1.25 (0.64, 2.43) 0.517
Q3	0.94 (0.50, 1.76) 0.851	1.15 (0.58, 2.28) 0.690	1.07 (0.54, 2.12) 0.855
Q4	2.42 (1.44, 4.06) 0.001	2.51 (1.40, 4.48) 0.002	2.24 (1.24, 4.03) 0.007

Model I covariates were adjusted for sex, age, race, and body mass index (BMI).

Model II covariates were adjusted for sex, age, race, BMI, heart rate, MAP, SpO_2_, myocardial infarction, congestive heart failure, peripheral vascular disease, cerebrovascular disease, chronic pulmonary disease, diabetes, renal disease, and cancer.

HR values less than 1 indicates a reduced risk of 90-day mortality.

**Figure 3:**
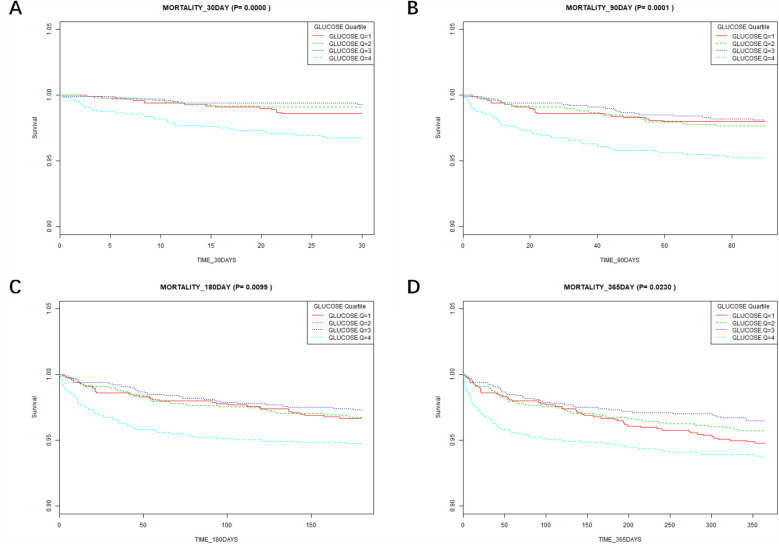
Results of Kaplan–Meier survival analysis at different time points. **(A)** Association between the glucose quartile and the 30-day mortality. **(B)** Association between the glucose quartile and the 90-day mortality. **(C)** Association between the glucose quartile and the 180-day mortality. **(D)** Association between the glucose quartile and the 365-day mortality.

According to the Kaplan–Meier survival curves (Figure 3), the low glucose groups (Q1–Q3) had a greater 30-day to 365-day survival probability than the high glucose group (Q4) (*P* < 0.05). This suggests that the high glucose group had a higher mortality.

### Associations between blood glucose and the lengths of hospital and ICU stays

In the fully adjusted model, taking blood glucose as a continuous variable, there was an association between each unit's rise in blood glucose and a 0.01-day increase in a hospital stay or a 0.01-day increase in ICU stay (Table 5).

**Table 5:** Univariate linear regression model to evaluate the associations between glucose and lengths of stay in the ICU and hospital.

Variables	Unadjusted		Model 1		Model 2	
	β (95% CI)	*P*-value	β (95% CI)	*P*-value	β (95% CI)	*P*-value
LOS ICU, days	0.01 (0.01, 0.02)	<0.001	0.02 (0.01, 0.02)	<0.001	0.01 (0.01, 0.02)	<0.001
LOS hospital, days	0.02 (0.02, 0.03)	<0.001	0.02 (0.02, 0.03)	<0.001	0.01 (0.00, 0.02)	0.003

Model 1 covariates were adjusted for sex, age, race, and body mass index (BMI).

Model 2 covariates were adjusted for sex, age, race, BMI, heart rate, MAP, SpO_2_, myocardial infarction, congestive heart failure, peripheral vascular disease, cerebrovascular disease, chronic pulmonary disease, diabetes, renal disease, and cancer.

LOS ICU, length of stay in ICU; LOS hospital, length of stay at hospital.

β: effect value. For every 1 mg/dl increase in blood glucose, ICU stay or hospitalization days increased by 0.01 days.

### The relationship between diabetes and 90-day mortality following ECC

To further validate our findings, we stratified the study population based on the presence or absence of diabetes. In the baseline analysis (Supplementary Table S2), the 90-day mortality rate was 2.54% in non-diabetic patients and 3.27% in diabetic patients (*P* = 0.187), indicating no statistically significant difference. In subgroup analysis (Supplementary Table S3), there was no statistically significant difference in mortality between diabetic and nondiabetic patients experiencing ECC 90 days after surgery in terms of sex, age, or comorbidities (*P* > 0.05). When stratified by blood glucose quartiles, the Q3 group showed a statistically significant association between diabetes and mortality (HR: 2.60, 95% CI:1.06–6.40, *P* < 0.05), with 10 out of 302 diabetic patients and 9 out of 700 nondiabetic patients dying in the Q3 group. This suggests a strong association between diabetic status and postoperative mortality in patients with blood glucose at the Q3 level. Notably, in the highest quartile of blood glucose (Q4), diabetic patients had a lower risk of death than non-diabetic patients (HR: 0.56, 95% CI:0.30–1.02, *P* = 0.058). Kaplan–Meier survival analysis for diabetic and non-diabetic patients indicated no significant difference between the groups (Supplementary Figure S1).

## Discussion

This research investigated the relationship between blood glucose and the 90-day mortality in 4,033 patients after adjusting for variables. Our research shows a significant connection between higher blood glucose levels and a higher 90-day mortality. Specifically, patients with higher blood glucose levels exhibited a markedly higher risk of mortality within 90 days post-operation compared to those with lower glucose levels. Notably, our RCS curve fitting analysis indicates that the risk of 90-day mortality progressively increases when blood glucose levels exceed 119 mg/dl, more stringent compared to the ≤126 mg/dl thresholds proposed by the American Diabetes Association (19). These findings highlight a potential threshold effect where higher blood glucose levels correlate with worse clinical outcomes. The identification of 119 mg/dl as a critical point may provide valuable insights for clinical practice. This threshold suggests that maintaining blood glucose levels below this value could be beneficial for reducing the risk of mortality in patients. The statistically significant association observed for blood glucose levels above this threshold underscores the need for careful monitoring and management of hyperglycemia to improve patient prognosis. Additionally, the relationship between blood glucose values and length of hospital stay and ICU stay showed that the higher the blood glucose levels, the correspondingly longer the length of hospital stay and ICU stay. Postoperative glucose monitoring and timely interventions for patients treated with ECC result in shorter hospital and ICU stays. This approach helps to reduce the financial burden on patients, promotes recovery, and facilitates early discharge (20). The Kaplan–Meier curve analysis indicated that postoperative blood glucose levels were associated with the all-cause mortality at 30–180 days. However, the strength of this association gradually diminished over time, suggesting that the long-term prognosis of patients is progressively less influenced by blood glucose levels following ECC.

Previous studies have demonstrated that postoperative glycemic variability in cardiac surgery patients independently impacts mortality, with postoperative hyperglycemia exerting a stronger adverse effect on survival than intraoperative hyperglycemia. Tight intraoperative glucose control below 200 mg/dl has been associated with reduced adverse events (21). A study focusing on coronary artery bypass grafting (CABG) reported that maintaining perioperative glucose levels below 140 mg/dl significantly lowered cardiovascular complications (22). Further investigation into the long-term outcomes of CABG patients revealed that those with perioperative glucose levels controlled under 150 mg/dl exhibited improved 5-year survival rates. Additionally, preoperative strict glycemic management has been shown to reduce postoperative complications in non-diabetic patients undergoing cardiac surgery (23).

Notably, subgroup analyses stratified by diabetic status (Supplementary Table S3) revealed a striking disparity: diabetic patients in the Q3 group demonstrated markedly higher mortality compared to non-diabetic counterparts, with a hazard ratio of 2.60. Paradoxically, in the Q4 group, diabetic patients exhibited a lower mortality risk (HR = 0.56) than non-diabetics, with a significant interaction effect (*P* for interaction <0.05), indicating differential impacts of glycemia on 90-day mortality between these populations. This aligns with evidence suggesting that non-diabetic patients experience worse cardiac surgical outcomes than diabetics under hyperglycemic conditions (24, 25), non-diabetic patients with postoperative blood glucose levels exceeding 250 mg/dl exhibited prolonged hospitalization duration and higher mortality rates compared to their diabetic counterparts (26).

A plausible explanation for this dichotomy may relate to enhanced glycemic tolerance in diabetic patients. Supporting this hypothesis, studies have observed that insulin-treated diabetic patients with postoperative glucose levels below 180 mg/dl paradoxically experienced prolonged hospitalization and increased complications, whereas non-insulin users—regardless of diabetic status—showed no significant difference in adverse events (20). This phenomenon may underlie the reduced mortality risk observed in Q4 group diabetics compared to their non-diabetic counterparts, potentially reflecting adaptive metabolic responses to chronic hyperglycemia in diabetic individuals. Chronic adaptation to hyperglycemia in diabetic patients may help mitigate the adverse effects of acute severe hyperglycemic fluctuations observed in nondiabetic patients.

Listed below are several potential causes of suboptimal postoperative glucose management. Several factors contribute to postoperative hyperglycemia, which in turn can increase mortality risk. Perioperative stress is a primary driver, leading to the release of stress hormones like catecholamines and cortisol (27, 28). These hormones promote gluconeogenesis and glycogenolysis, resulting in elevated blood glucose levels (29, 30). Additionally, surgical trauma can induce insulin resistance, further exacerbating hyperglycemia. This process also involves releasing pro-inflammatory factors such as *IL-6* and *TNF-*α (31–33). These cytokines impair insulin signaling pathways and contribute to the onset of insulin resistance. In a simulated ECC model, it was found that hemolysis promotes insulin degradation. The greater the degree of hemolysis, the more insulin is degraded. This suggests that hemolysis during ECC in a clinical setting may lead to elevated blood glucose levels in patients (8).

Perioperative blood glucose levels have significant implications for patient outcomes and can lead to adverse events. Elevated blood glucose levels are strongly associated with endothelial dysfunction, which is crucial in the pathogenesis of cardiovascular complications (34). This dysfunction not only heightens the risk of thrombotic events but also impairs wound healing, increasing the susceptibility to infections and sepsis. These complications collectively contribute to the observed increase in mortality rates among patients with hyperglycemia. Additionally, stress-induced hyperglycemia can precipitate severe metabolic disturbances, such as diabetic ketoacidosis and hyperosmolar hyperglycemic state, both of which are linked to elevated mortality. Persistent hyperglycemia may also reflect underlying metabolic dysregulation and serve as a marker of adverse patient prognosis. Greet Van has indicated that maintaining blood glucose at 110 mg/dl can reduce mortality from multi-organ failure in ICU patients with sepsis, regardless of their diabetic status, and can also decrease the incidence of other complications (35). Additionally, some scholars have posited that hyperglycemia during ECC may increase the risk of neurological injury, potentially due to the heightened risk of cerebral ischemia associated with elevated blood glucose levels (36). After ECC, the patient is admitted to the ICU and is in a state of mechanical ventilation and fasting, and hypoglycemia may also occur. Acquired long QT syndrome can be caused by hypoglycemia and can also lead to adverse events similar to patient fainting (37, 38). Thus, hyperglycemia and hypoglycemia management in the perioperative period should be considered a critical factor in patient management, warranting vigilant monitoring and intervention to mitigate its impact on mortality and morbidity.

This study has several strengths. First, the use of large, well-defined cohorts improves the generalizability of our findings. Second, the comprehensiveness of the dataset allows us to control for various confounding variables, strengthening the results' robustness. Numerous discoveries have shown that age, BMI, potassium, creatinine, lymphocytes, ventilation hours, complications such as CHF, RD, and other risk factors can also have a substantial effect on patient mortality after cardiac surgery (39–42). In the present study, these factors were well-controlled. Furthermore, the retrospective design enabled us to analyze real-world data over an extended period, providing valuable insights into long-term outcomes.

However, our research has some limitations. First, retrospective cohort design inherently carries the risk of selection bias and unmeasured confounding. Although, we adjusted for numerous potentially influential covariates; however, residual confounders could not be completely eliminated. Second, our study relied on the accuracy and completeness of electronic medical records, which may be subject to documentation errors. For example, hemoglobin a1c was recorded in the dataset; however, due to a missing rate exceeding 30%, which could affect the accuracy of the statistical results, we did not include hemoglobin a1c in the analysis. Third, the monocentric nature of this study may limit the generalizability of our findings. Future research should consider conducting similar investigations in a multicenter cohort to enhance external validity. Additionally, there were relatively few patients with hypoglycemia in our study database, with only 40 patients with blood glucose <70 mg/dl, so we could not determine the lowest value of the patient's blood glucose levels. We used the lower limit of blood glucose values (80 mg/dl) in the surgical ICU reported by other studies as a reference (10, 35). Furthermore, preoperative and intraoperative blood glucose levels were not considered, which may have influenced our findings. Finally, we excluded patients requiring repeat cardiac surgery, potentially introducing selection bias and affecting the generalizability of our results.

## Conclusion

According to our research, blood glucose levels were nonlinearly associated with 90-day mortality after ECC. When blood glucose levels exceeded 119 mg/dl, blood glucose levels were positively associated with 90-day postoperative mortality. Blood glucose is an accessible and convenient laboratory test that can provide early postoperative screening as a risk factor for a poor prognosis. As a result, blood glucose levels could be clinically meaningful and be employed extensively in the future.

## Data Availability

The datasets presented in this study can be found in online repositories. The names of the repository/repositories and accession number(s) can be found in the article/Supplementary Material.

## References

[1] McGuinness SP Parke RL Drummond K Willcox T Bailey M Kruger C A multicenter, randomized, controlled phase IIb trial of avoidance of hyperoxemia during cardiopulmonary bypass. Anesthesiology. (2016) 125:465–73. 10.1097/ALN.0000000000001226 27404222

[2] von Loeffelholz C Birkenfeld AL. Tight versus liberal blood-glucose control in the intensive care unit: special considerations for patients with diabetes. Lancet Diabetes Endocrinol. (2024) 12:277–84. 10.1016/S2213-8587(24)00058-5 38514241

[3] Fowler AJ Wahedally MAH Abbott TEF Smuk M Prowle JR Pearse RM Death after surgery among patients with chronic disease: prospective study of routinely collected data in the english NHS. Br J Anaesth. (2022) 128:333–42. 10.1016/j.bja.2021.11.011 34949439

[4] Ketan D Dileep L Agnes G Mike G Clare H Andrea L Guideline for Perioperative Care for People with Diabetes Mellitus Undergoing Elective and Emergency Surgery. London: Centre for Perioperative Care (CPOC) (2021).

[5] Chen W Yu P Chen C Cai S Chen J Zheng C Association between the red blood cell distribution width and 30-day mortality in intensive care patients undergoing cardiac surgery: a retrospective observational study based on the medical information mart for intensive care-IV database. Ann Lab Med. (2024) 44:401–9. 10.3343/alm.2023.0345 38469636 PMC11169773

[6] Lazam S Vanoverschelde J-L Tribouilloy C Grigioni F Suri RM Avierinos J-F Twenty-year outcome after mitral repair versus replacement for severe degenerative mitral regurgitation: analysis of a large, prospective, multicenter, international registry. Circulation. (2017) 135:410–22. 10.1161/CIRCULATIONAHA.116.023340 27899396

[7] Umpierrez GE Pasquel FJ. Management of inpatient hyperglycemia and diabetes in older adults. Diabetes Care. (2017) 40:509–17. 10.2337/dc16-0989 28325798 PMC5864102

[8] Schweizer T Nossen CM Galova B Schild C Huber M Bally L *In vitro* investigation of insulin dynamics during 4 hours of simulated cardiopulmonary bypass. Anesth Analg. (2024). 10.1213/ANE.0000000000007106 38861464 PMC12220572

[9] Anderson RE Brismar K Barr G Ivert T. Effects of cardiopulmonary bypass on glucose homeostasis after coronary artery bypass surgery. Eur J Cardiothorac Surg. (2005) 28:425–30. 10.1016/j.ejcts.2005.05.025 16054822

[10] Pasquel FJ Lansang MC Dhatariya K Umpierrez GE. Management of diabetes and hyperglycaemia in the hospital. Lancet Diabetes Endocrinol. (2021) 9:174–88. 10.1016/S2213-8587(20)30381-8 33515493 PMC10423081

[11] Philis-Tsimikas A Asong M Franek E Jia T Rosenstock J Stachlewska K Switching to once-weekly insulin icodec versus once-daily insulin degludec in individuals with basal insulin-treated type 2 diabetes (ONWARDS 2): a phase 3a, randomised, open label, multicentre, treat-to-target trial. Lancet Diabetes Endocrinol. (2023) 11:414–25. 10.1016/S2213-8587(23)00093-1 37148899

[12] Cha S-A Yun J-S Kim G-H Ahn Y-B. Impact of hypoglycemia at the time of hospitalization for heart failure from emergency department on major adverse cardiovascular events in patients with and without type 2 diabetes. Cardiovasc Diabetol. (2022) 21:218. 10.1186/s12933-022-01651-0 36271363 PMC9585717

[13] Partridge H Perkins B Mathieu S Nicholls A Adeniji K. Clinical recommendations in the management of the patient with type 1 diabetes on insulin pump therapy in the perioperative period: a primer for the anaesthetist. Br J Anaesth. (2016) 116:18–26. 10.1093/bja/aev347 26675948

[14] Gandhi GY Nuttall GA Abel MD Mullany CJ Schaff HV O’Brien PC Intensive intraoperative insulin therapy versus conventional glucose management during cardiac surgery: a randomized trial. Ann Intern Med. (2007) 146:233–43. 10.7326/0003-4819-146-4-200702200-00002 17310047

[15] Johnson AEW Bulgarelli L Shen L Gayles A Shammout A Horng S MIMIC-IV, a freely accessible electronic health record dataset. Sci Data. (2023) 10:1. 10.1038/s41597-022-01899-x 36596836 PMC9810617

[16] Goldberger AL Amaral LA Glass L Hausdorff JM Ivanov PC Mark RG Physiobank, PhysioToolkit, and PhysioNet: components of a new research resource for complex physiologic signals. Circulation. (2000) 101:E215–220. 10.1161/01.cir.101.23.e215 10851218

[17] Yang Z Gong H Kan F Ji N. Association between the triglyceride glucose (TyG) index and the risk of acute kidney injury in critically ill patients with heart failure: analysis of the MIMIC-IV database. Cardiovasc Diabetol. (2023) 22:232. 10.1186/s12933-023-01971-9 37653418 PMC10472684

[18] Chen C Dai J-L. Triglyceride to high-density lipoprotein cholesterol (HDL-C) ratio and arterial stiffness in Japanese population: a secondary analysis based on a cross-sectional study. Lipids Health Dis. (2018) 17:130. 10.1186/s12944-018-0776-7 29843793 PMC5975424

[19] American Diabetes Association. 2. Classification and diagnosis of diabetes: standards of medical care in diabetes-2019. Diabetes Care. (2019) 42:S13–28. 10.2337/dc19-S002 30559228

[20] Greco G Ferket BS D’Alessandro DA Shi W Horvath KA Rosen A Diabetes and the association of postoperative hyperglycemia with clinical and economic outcomes in cardiac surgery. Diabetes Care. (2016) 39:408–17. 10.2337/dc15-1817 26786574 PMC4764032

[21] Duncan AE Abd-Elsayed A Maheshwari A Xu M Soltesz E Koch CG. Role of intraoperative and postoperative blood glucose concentrations in predicting outcomes after cardiac surgery. Anesthesiology. (2010) 112:860–71. 10.1097/ALN.0b013e3181d3d4b4 20216389

[22] Chen Y Zhang H Hou X Li X Qian X Feng X Glycemic control and risk factors for in-hospital mortality and vascular complications after coronary artery bypass grafting in patients with and without preexisting diabetes. J Diabetes. (2021) 13:232–42. 10.1111/1753-0407.13108 32833247 PMC7891320

[23] Mansur A Popov AF Hanna AA Bergmann I Brandes IF Beissbarth T Bauer M Hinz J. Perioperative blood glucose levels <150 mg/dl are associated with improved 5-year survival in patients undergoing on-pump cardiac surgery: a prospective, observational cohort study. Medicine (Baltimore) (2015) 94:e2035. 10.1097/MD.0000000000002035 26559310 PMC4912304

[24] Egi M Bellomo R Stachowski E French CJ Hart GK Hegarty C Blood glucose concentration and outcome of critical illness: the impact of diabetes. Crit Care Med. (2008) 36:2249–55. 10.1097/CCM.0b013e318181039a 18664780

[25] Sechterberger MK Bosman RJ Oudemans-van Straaten HM Siegelaar SE Hermanides J Hoekstra JB The effect of diabetes mellitus on the association between measures of glycaemiccontrol and ICU mortality: a retrospective cohort study. Crit Care. (2013) 17:R52. 10.1186/cc12572 23510051 PMC3733428

[26] Székely A Levin J Miao Y Tudor IC Vuylsteke A Ofner P Impact of hyperglycemia on perioperative mortality after coronary artery bypass graft surgery. J Thorac Cardiovasc Surg. (2011) 142:430–437.e1. 10.1016/j.jtcvs.2011.03.009 21497835

[27] Barth E Albuszies G Baumgart K Matejovic M Wachter U Vogt J Glucose metabolism and catecholamines. Crit Care Med. (2007) 35:S508–518. 10.1097/01.CCM.0000278047.06965.20 17713401

[28] Palermo NE Gianchandani RY McDonnell ME Alexanian SM. Stress hyperglycemia during surgery and anesthesia: pathogenesis and clinical implications. Curr Diab Rep. (2016) 16:33. 10.1007/s11892-016-0721-y 26957107

[29] Losser M-R Damoisel C Payen D. Bench-to-bedside review: glucose and stress conditions in the intensive care unit. Crit Care. (2010) 14:231. 10.1186/cc9100 20727232 PMC2945096

[30] Zauner A Nimmerrichter P Anderwald C Bischof M Schiefermeier M Ratheiser K Severity of insulin resistance in critically ill medical patients. Metab Clin Exp. (2007) 56:1–5. 10.1016/j.metabol.2006.08.014 17161218

[31] McDonnell ME Umpierrez GE. Insulin therapy for the management of hyperglycemia in hospitalized patients. Endocrinol Metab Clin North Am. (2012) 41:175–201. 10.1016/j.ecl.2012.01.001 22575413 PMC3738170

[32] Zakrzewski D Janas J Heretyk H Stepińska J. Inflammatory response and postoperative kidney failure in patients with diabetes type 2 or impaired glucose tolerance undergoing heart valve surgery. Kardiol Pol. (2010) 68:530–6. 10.33963/v.kp.79735 20491014

[33] Zhao G Cao S Cui J. Fast-track surgery improves postoperative clinical recovery and reduces postoperative insulin resistance after esophagectomy for esophageal cancer. Support Care Cancer. (2014) 22:351–8. 10.1007/s00520-013-1979-0 24068549

[34] Chen Q Jiang D Shan Z. The influence of dipeptidyl peptidase-4 inhibitor on the progression of type B intramural hematoma. Front Cardiovasc Med. (2022) 9:969357. 10.3389/fcvm.2022.969357 36330007 PMC9623157

[35] van den Berghe G Wouters P Weekers F Verwaest C Bruyninckx F Schetz M Intensive insulin therapy in critically ill patients. N Engl J Med. (2001) 345:1359–67. 10.1056/NEJMoa011300 11794168

[36] Lanier WL. Glucose management during cardiopulmonary bypass: cardiovascular and neurologic implications. Anesth Analg. (1991) 72:423–7. 10.1213/00000539-199104000-00002 2006732

[37] Robinson RTCE Harris ND Ireland RH Lee S Newman C Heller SR. Mechanisms of abnormal cardiac repolarization during insulin-induced hypoglycemia. Diabetes. (2003) 52:1469–74. 10.2337/diabetes.52.6.1469 12765959

[38] Boucai L Southern WN Zonszein J. Hypoglycemia-associated mortality is not drug-associated but linked to comorbidities. Am J Med. (2011) 124:1028–35. 10.1016/j.amjmed.2011.07.011 22017781 PMC3200530

[39] Liu X Xie L Zhu W Zhou Y. Association of body mass index and all-cause mortality in patients after cardiac surgery: a dose-response meta-analysis. Nutrition. (2020) 72:110696. 10.1016/j.nut.2019.110696 32007807

[40] Zante B Reichenspurner H Kubik M Kluge S Schefold JC Pfortmueller CA. Base excess is superior to lactate-levels in prediction of ICU mortality after cardiac surgery. PLoS One. (2018) 13:e0205309. 10.1371/journal.pone.0205309 30289956 PMC6173442

[41] Yu Y Peng C Zhang Z Shen K Zhang Y Xiao J Machine learning methods for predicting long-term mortality in patients after cardiac surgery. Front Cardiovasc Med. (2022) 9:831390. 10.3389/fcvm.2022.831390 35592400 PMC9110683

[42] Bouma HR Mungroop HE Scheeren TWL Epema AH. Very early creatinine changes and 30-day mortality after cardiac surgery. Eur J Anaesthesiol. (2021) 38:665. 10.1097/EJA.0000000000001436 33967257

