# Supplementary figures and images for: The relationship between blood glucose and clinical outcomes after extracorporeal circulation: a retrospective cohort study
Source: Front Cardiovasc Med. 2025 Mar 31;12:1480163. doi: 10.3389/fcvm.2025.1480163 (PMC11994716; doi:10.3389/fcvm.2025.1480163)

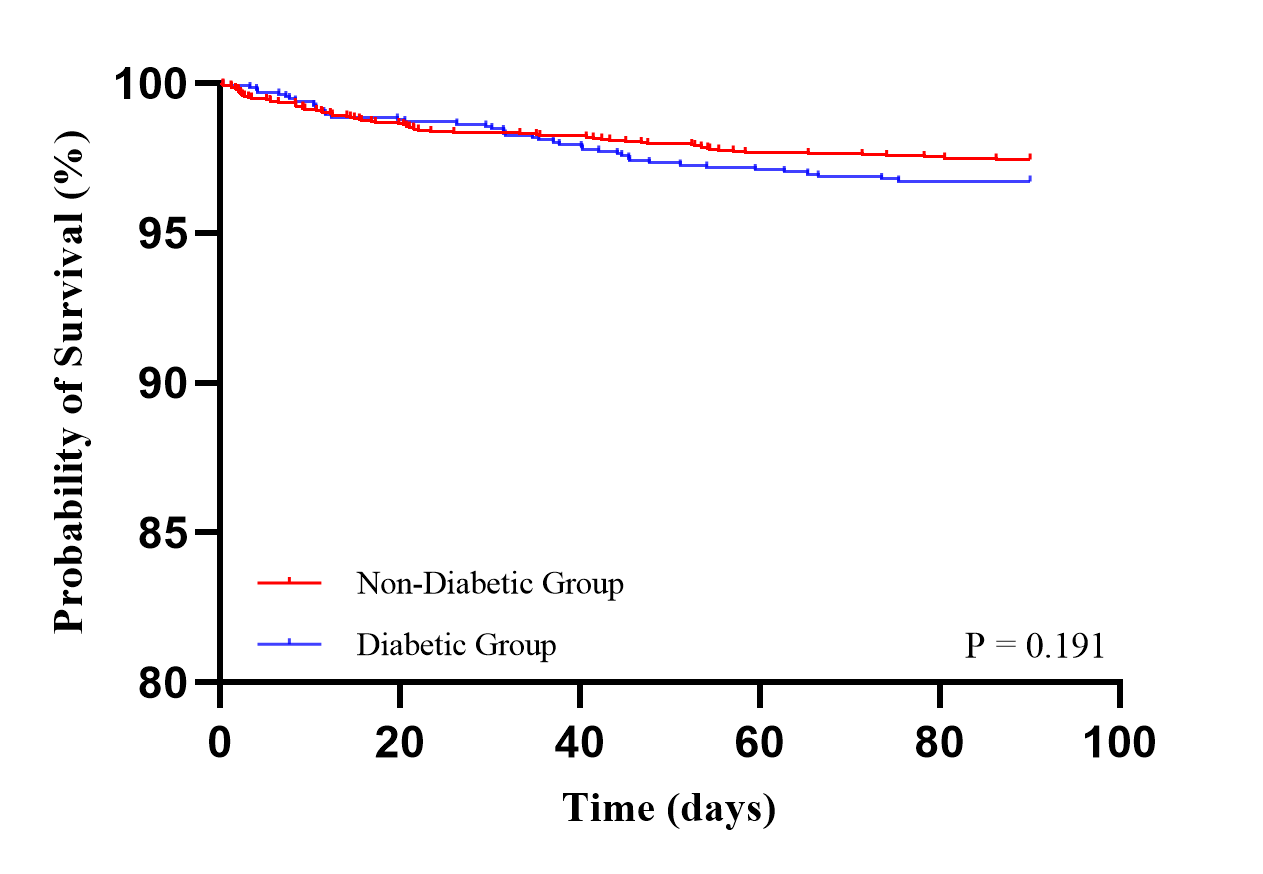

Supplement: Supplementary file 1 [file Image1.tif]
