# Supplementary material for: The relationship between blood glucose and clinical outcomes after extracorporeal circulation: a retrospective cohort study
Source: Front Cardiovasc Med. 2025 Mar 31;12:1480163. doi: 10.3389/fcvm.2025.1480163 (PMC11994716; doi:10.3389/fcvm.2025.1480163)
Supplement: Supplementary file 2 [file Table1.docx]

**Supplementary Table S1 Association between postoperative blood glucose and outcomes.**

| Blood Glucose Quartile | Q1 | Q2 | Q3 | Q4 | P-value |
| --- | --- | --- | --- | --- | --- |
| Overall (n, %) | 996 (24.70%) | 986 (24.45%) | 1002 (24.85%) | 1049 (26.01%) |  |
| Mortality 30-day (%) |  |  |  |  | <0.001 |
| No | 98.6 | 99.1 | 99.3 | 96.8 |  |
| Yes | 1.4 | 0.9 | 0.7 | 3.2 |  |
| Mortality 60-day (%) |  |  |  |  | <0.001 |
| No | 98.0 | 98.0 | 98.5 | 95.6 |  |
| Yes | 2.0 | 2.0 | 1.5 | 4.4 |  |
| Mortality 90-day (%) |  |  |  |  | <0.001 |
| No | 98.0 | 97.7 | 98.1 | 95.2 |  |
| Yes | 2.0 | 2.3 | 1.9 | 4.8 |  |
| Mortality 180-day (%) |  |  |  |  | 0.012 |
| No | 96.7 | 96.8 | 97.3 | 94.8 |  |
| Yes | 3.3 | 3.2 | 2.7 | 5.2 |  |
| Mortality 365-day (%) |  |  |  |  | 0.027 |
| No | 94.8 | 95.7 | 96.5 | 93.8 |  |
| Yes | 5.2 | 4.3 | 3.5 | 6.2 |  |
