# Supplementary material for: The relationship between blood glucose and clinical outcomes after extracorporeal circulation: a retrospective cohort study
Source: Front Cardiovasc Med. 2025 Mar 31;12:1480163. doi: 10.3389/fcvm.2025.1480163 (PMC11994716; doi:10.3389/fcvm.2025.1480163)
Supplement: Supplementary file 3 [file Table2.docx]

**Supplementary Table S2 Baseline characteristics of the study participants.**

| Variables | Non-Diabetic Group (n=2717) | Diabetic Group (n=1316) | HR (95%CI) | P-value |
| --- | --- | --- | --- | --- |
| Age, years | 67.31 ± 12.83 | 68.84 ± 10.01 | 0.13 (0.07, 0.20) | <0.001 |
| Gender, sex |  |  | 0.03 (-0.03, 0.10) | 0.320 |
| Male, n (%) | 1904 (70.08%) | 902 (68.54%) |  |  |
| Female, n (%) | 813 (29.92%) | 414 (31.46%) |  |  |
| Race |  |  | 0.15 (0.08, 0.21) | <0.001 |
| Asian, n (%) | 59 (2.17%) | 27 (2.05%) |  |  |
| Black, n (%) | 75 (2.76%) | 75 (5.70%) |  |  |
| White, n (%) | 2136 (78.62%) | 998 (75.84%) |  |  |
| Unknown, n (%) | 354 (13.03%) | 167 (12.69%) |  |  |
| Other, n (%) | 93 (3.42%) | 49 (3.72%) |  |  |
| BMI, kg/m2 | 30.23 ± 5.91 | 32.85 ± 6.46 | 0.42 (0.35, 0.49) | <0.001 |
| Heart Rate, bpm | 80.96 ± 11.47 | 81.33 ± 10.88 | 0.03 (-0.03, 0.10) | 0.333 |
| MAP (mmHg) | 71.12 ± 12.20 | 71.50 ± 12.44 | 0.03 (-0.04, 0.10) | 0.358 |
| SBP (mmHg) | 110.48 ± 17.72 | 114.82 ± 19.81 | 0.23 (0.16, 0.30) | <0.001 |
| DBP (mmHg) | 59.35 ± 12.43 | 58.38 ± 12.25 | 0.08 (0.01, 0.14) | 0.021 |
| SpO_2_ (%) | 96.23 ± 5.97 | 96.30 ± 5.40 | 0.01 (-0.06, 0.09) | 0.776 |
| Glucose (mg/dL) | 119.16 ± 29.93 | 131.36 ± 50.88 | 0.29 (0.23, 0.36) | <0.001 |
| Potassium (mmol/L) | 4.20 ± 0.52 | 4.26 ± 0.52 | 0.12 (0.05, 0.18) | <0.001 |
| Creatinine (mg/dL) | 0.94 ± 0.66 | 1.13 ± 0.94 | 0.23 (0.16, 0.29) | <0.001 |
| Hemoglobin (g/dL) | 10.15 ± 2.08 | 9.96 ± 2.06 | 0.09 (0.02, 0.15) | 0.008 |
| Neutrophils (10^9/L) | 9.79 ± 5.00 | 9.25 ± 4.16 | 0.12 (0.01, 0.22) | 0.036 |
| Monocytes (10^9/L) | 0.38 ± 0.28 | 0.37 ± 0.25 | 0.03 (-0.07, 0.14) | 0.582 |
| Lymphocytes (10^9/L) | 2.06 ± 3.96 | 2.07 ± 1.05 | 0.00 (-0.10, 0.11) | 0.945 |
| WBCs (10^9/L) | 12.26 ± 5.85 | 11.87 ± 5.05 | 0.07 (0.00, 0.14) | 0.041 |
| Ventilation Hours (hours) | 60.51 ± 105.81 | 61.55 ± 95.41 | 0.01 (-0.06, 0.08) | 0.764 |
| LOS ICU (days) | 2.95 ± 4.04 | 3.03 ± 3.99 | 0.02 (-0.04, 0.09) | 0.508 |
| LOS hospital (days) | 8.61 ± 6.05 | 9.42 ± 7.35 | 0.12 (0.06, 0.19) | <0.001 |
| Myocardial Infarct |  |  | 0.29 (0.22, 0.36) | <0.001 |
| No, n (%) | 2129 (78.36%) | 861 (65.43%) |  |  |
| Yes, n (%) | 588 (21.64%) | 455 (34.57%) |  |  |
| Congestive Heart Failure |  |  | 0.15 (0.08, 0.21) | <0.001 |
| No, n (%) | 2057 (75.71%) | 911 (69.22%) |  |  |
| Yes, n (%) | 660 (24.29%) | 405 (30.78%) |  |  |
| Peripheral Vascular Disease |  |  | 0.03 (-0.03, 0.10) | 0.320 |
| No, n (%) | 2258 (83.11%) | 1110 (84.35%) |  |  |
| Yes, n (%) | 459 (16.89%) | 206 (15.65%) |  |  |
| Cerebrovascular Disease |  |  | 0.11 (0.04, 0.17) | 0.001 |
| No, n (%) | 2457 (90.43%) | 1146 (87.08%) |  |  |
| Yes, n (%) | 260 (9.57%) | 170 (12.92%) |  |  |
| Chronic Pulmonary Disease |  |  | 0.03 (-0.03, 0.10) | 0.313 |
| No, n (%) | 2050 (75.45%) | 1012 (76.90%) |  |  |
| Yes, n (%) | 667 (24.55%) | 304 (23.10%) |  |  |
| Renal Disease |  |  | 0.33 (0.27, 0.40) | <0.001 |
| No, n (%) | 2448 (90.10%) | 1027 (78.04%) |  |  |
| Yes, n (%) | 269 (9.90%) | 289 (21.96%) | . |  |
| Cancer |  |  | 0.07 (0.01, 0.14) | 0.025 |
| No, n (%) | 2650 (97.53%) | 1267 (96.28%) |  |  |
| Yes, n (%) | 67 (2.47%) | 49 (3.72%) |  |  |
| 90-Day Mortality |  |  | 0.04 (-0.02, 0.11) | 0.187 |
| No, n (%) | 2648 (97.46%) | 1273 (96.73%) |  |  |
| Yes, n (%) | 69 (2.54%) | 43 (3.27%) |  |  |

Abbreviations: HR: Hazard Ratio, CI: Confidence Interval.

HR values less than 1 indicates a reduced risk of 90-day mortality.
