# Supplementary material for: The relationship between blood glucose and clinical outcomes after extracorporeal circulation: a retrospective cohort study
Source: Front Cardiovasc Med. 2025 Mar 31;12:1480163. doi: 10.3389/fcvm.2025.1480163 (PMC11994716; doi:10.3389/fcvm.2025.1480163)
Supplement: Supplementary file 4 [file Table3.docx]

**Supplementary Table S3 Subgroup analysis of 90-day mortality in diabetic and nondiabetic patients.**

| Variables | Overall | Non-Diabetic Group | Diabetic Group | HR (95%CI) | *P* | P for interaction |
| --- | --- | --- | --- | --- | --- | --- |
|  |  |  |  |  |  |  |
| Overall | 4033 (100.00) | 69/2717 | 43/1316 | 1.29 (0.88 - 1.89) | 0.193 |  |
| Gender |  |  |  |  |  | 0.594 |
| Male | 2806 (69.58) | 41/1904 | 27/902 | 1.39 (0.86 - 2.26) | 0.183 |  |
| Female | 1227 (30.42) | 28/813 | 16/414 | 1.12 (0.61 - 2.08) | 0.710 |  |
| Myocardial Infarct |  |  |  |  |  | 0.695 |
| No | 2990 (74.14) | 45/2129 | 23/861 | 1.26 (0.76 - 2.09) | 0.363 |  |
| Yes | 1043 (25.86) | 24/588 | 20/455 | 1.08 (0.60 - 1.95) | 0.801 |  |
| Congestive Heart Failure |  |  |  |  |  | 0.630 |
| No | 2968 (73.59) | 36/2057 | 17/911 | 1.07 (0.60 - 1.90) | 0.830 |  |
| Yes | 1065 (26.41) | 33/660 | 26/405 | 1.29 (0.77 - 2.15) | 0.335 |  |
| Peripheral Vascular Disease |  |  |  |  |  | 0.197 |
| No | 3368 (83.51) | 42/2258 | 32/1110 | 1.56 (0.98 - 2.47) | 0.059 |  |
| Yes | 665 (16.49) | 27/459 | 11/206 | 0.90 (0.44 - 1.81) | 0.761 |  |
| Cerebrovascular Disease |  |  |  |  |  | 0.569 |
| No | 3603 (89.34) | 54/2457 | 33/1146 | 1.31 (0.85 - 2.02) | 0.218 |  |
| Yes | 430 (10.66) | 15/260 | 10/170 | 1.01 (0.45 - 2.24) | 0.985 |  |
| Chronic Pulmonary Disease |  |  |  |  |  | 0.842 |
| No | 3062 (75.92) | 45/2050 | 28/1012 | 1.26 (0.79 - 2.02) | 0.332 |  |
| Yes | 971 (24.08) | 24/667 | 15/304 | 1.37 (0.72 - 2.61) | 0.339 |  |
| Renal Disease |  |  |  |  |  | 0.923 |
| No | 3475 (86.16) | 51/2448 | 23/1027 | 1.07 (0.66 - 1.76) | 0.777 |  |
| Yes | 558 (13.84) | 18/269 | 20/289 | 1.03 (0.55 - 1.95) | 0.923 |  |
| Cancer |  |  |  |  |  | 0.992 |
| No | 3917 (97.12) | 67/2650 | 43/1267 | 1.34 (0.92 - 1.97) | 0.130 |  |
| Yes | 116 (2.88) | 2/67 | 0/49 | 0.00 (0.00 - Inf) | 0.999 |  |
| Age |  |  |  |  |  | 0.226 |
| <70 | 2208 (54.75) | 32/1510 | 14/698 | 0.94 (0.50 - 1.77) | 0.855 |  |
| ≥70 | 1825 (45.25) | 37/1207 | 29/618 | 1.54 (0.95 - 2.51) | 0.081 |  |
| Blood Glucose Quantile |  |  |  |  |  | 0.010 |
| Q1 | 996 (24.70) | 11/688 | 9/308 | 1.84 (0.76 - 4.44) | 0.174 |  |
| Q2 | 986 (24.45) | 14/732 | 9/254 | 1.86 (0.80 - 4.29) | 0.148 |  |
| Q3 | 1002 (24.85) | 9/700 | 10/302 | 2.60 (1.06 - 6.40) | 0.037 |  |
| Q4 | 1049 (26.01) | 35/597 | 15/452 | 0.56 (0.30 - 1.02) | 0.058 |  |
| Abbreviations: HR: Hazard Ratio, CI: Confidence Interval, Inf: Infinity.  HR values less than 1 indicates a reduced risk of 90-day mortality. | | | | | | |
